# Supplementary figures and images for: Mitochondria-targeted triphenylphosphonium-based compounds do not affect estrogen receptor α
Source: PeerJ. 2020 Mar 25;8:e8803. doi: 10.7717/peerj.8803 (PMC7102506; doi:10.7717/peerj.8803)

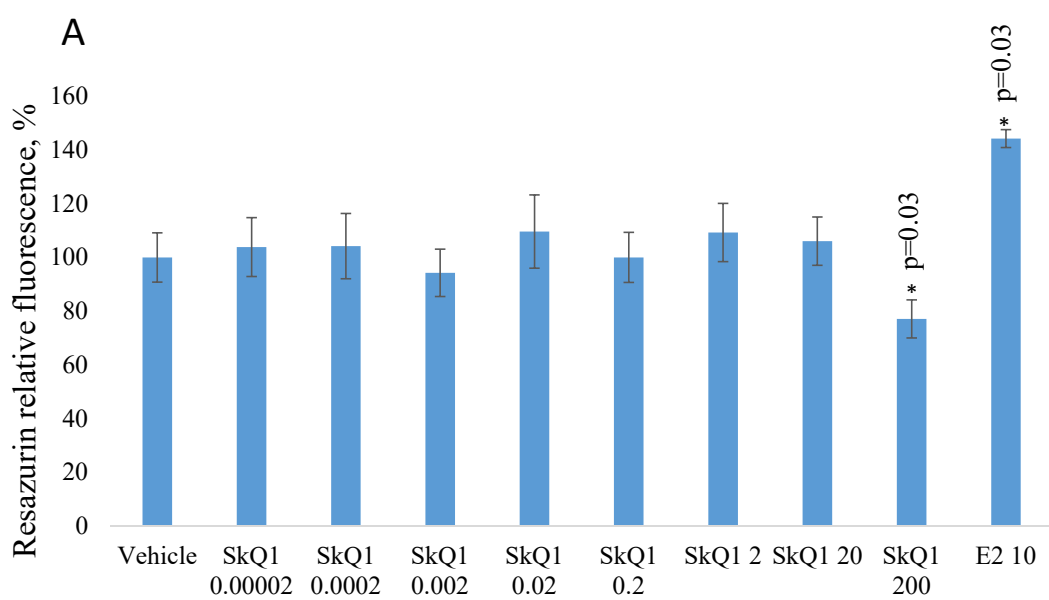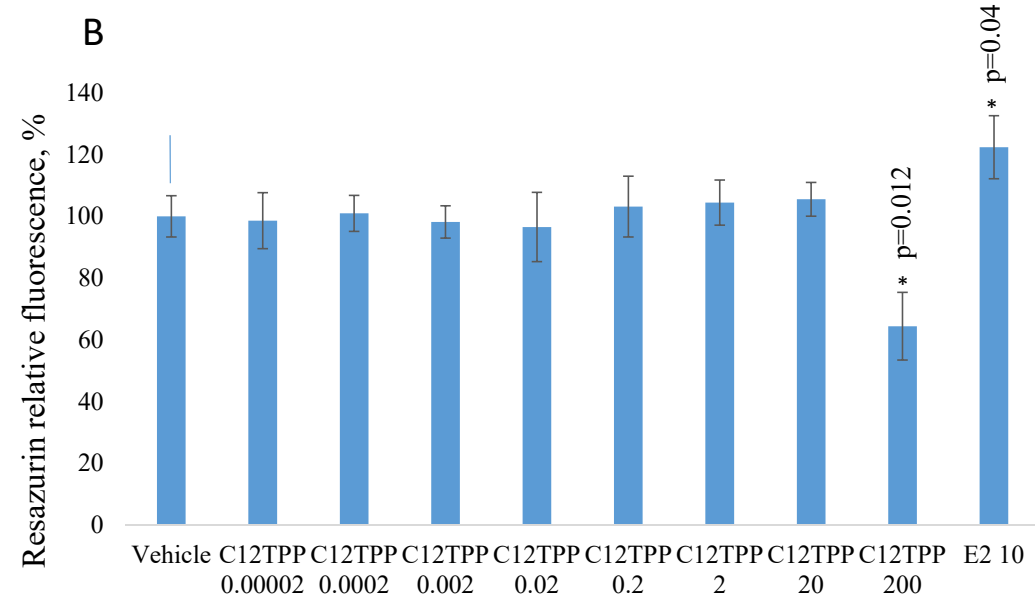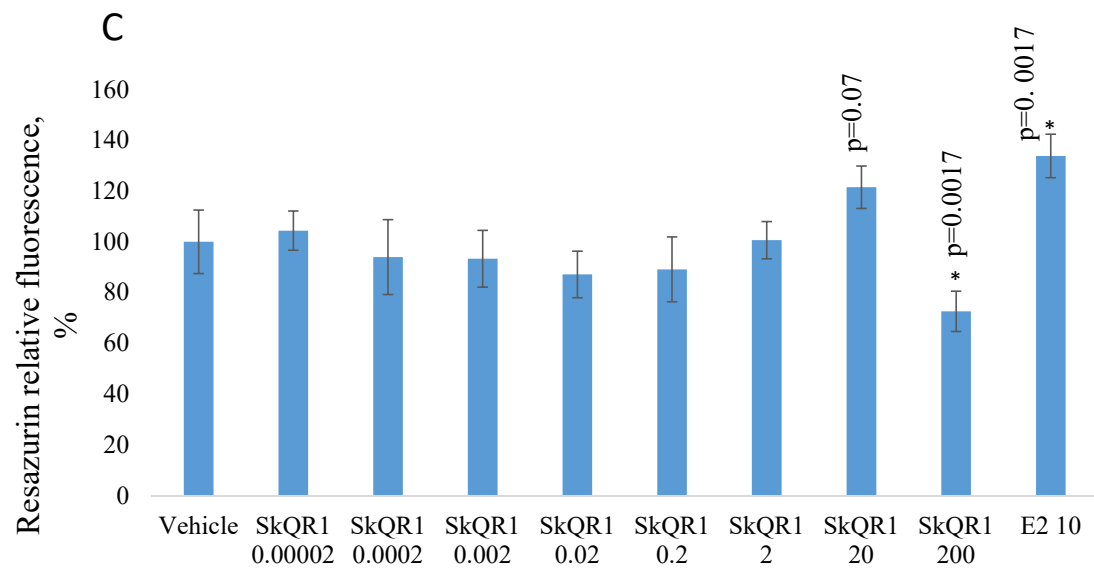

Supplement: Figure S1 — MCF-7 cells (approximately 5,000 cells per well) were incubated for 48 h with the test compounds at the indicated (nM) concentrations. (A) SkQ1, (B) C12 TPP, and (C) SkQR1. Cell viability was measured according to resazurin fluorescence. Data are expressed as the percentage of the control untreated cells +/ − SD, n = 5 technical replicates. The p-values were determined using unpaired Wilcoxon tests with Bonferroni correction for multiple measurements compared with the untreated control (Vehicle). [file peerj-08-8803-s007.pdf]

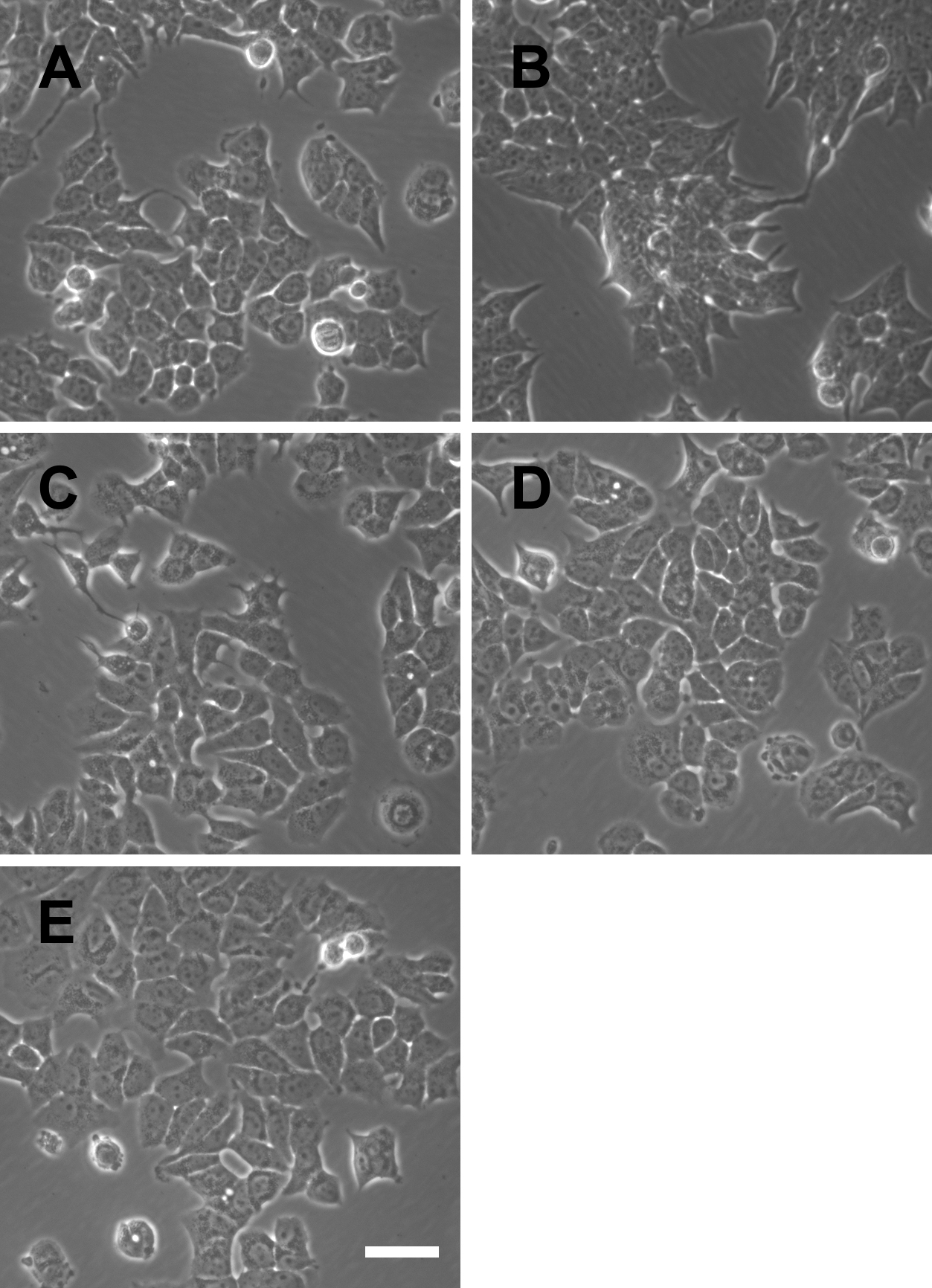

Supplement: Figure S2 — MCF-7 cells were treated as indicated in Fig. 4, and images were acquired using phase-contrast microscopy after 72 h. (A) Control cells. (B) 10 nM E2. (C) 20 nM SkQ1. (D) 20 nM C_12TPP. (E) 20 nM SkQR1. Bar, 50 µm. [file peerj-08-8803-s008.jpg]
